# Progress, applications, and challenges in high-throughput effect-directed analysis for toxicity driver identification — is it time for HT-EDA?

**DOI:** 10.1007/s00216-024-05424-4

**Published:** 2024-07-12

**Authors:** Iker Alvarez-Mora, Katarzyna Arturi, Frederic Béen, Sebastian Buchinger, Abd El Rahman El Mais, Christine Gallampois, Meike Hahn, Juliane Hollender, Corine Houtman, Sarah Johann, Martin Krauss, Marja Lamoree, Maria Margalef, Riccardo Massei, Werner Brack, Melis Muz

**Affiliations:** 1 Department of Exposure Science, Helmholtz Centre for Environmental Research, UFZ, Leipzig, Germany; 2 Research Centre for Experimental Marine Biology and Biotechnology (PIE), University of the Basque Country (UPV/EHU), Plentzia, Basque Country Spain; 3 Eawag, Swiss Federal Institute of Aquatic Science and Technology, Dübendorf, Switzerland; 4 KWR Water Research Institute, Nieuwegein, the Netherlands; 5 Chemistry for Environment and Health, Amsterdam Institute for Life and Environment (A-LIFE), Vrije Universiteit Amsterdam, Amsterdam, the Netherlands; 6 Department of Biochemistry and Ecotoxicology, Federal Institute of Hydrology (BfG), Koblenz, Germany; 7 Ineris, Parc Technologique Alata, Verneuil-en-Halatte, France; 8 Department of Chemistry, Umeå University, Umeå, Sweden; 9 Institute of Biogeochemistry and Pollutant Dynamics, ETH Zurich, Zürich, Switzerland; 10 The Water Laboratory, Haarlem, the Netherlands; 11 Department of Evolutionary Ecology and Environmental Toxicology, Goethe University Frankfurt, Frankfurt Am Main, Germany; 12 Department of Monitoring and Exploration Technologies, Research Data Management Team (RDM), Helmholtz Centre for Environmental Research, UFZ, Leipzig, Germany; 13 Department of Ecotoxicology, Group of Integrative Toxicology (iTox), Helmholtz Centre for Environmental Research, UFZ, Leipzig, Germany

**Keywords:** HT-EDA, NTS, Bioanalytical methods, Mass spectrometry

## Abstract

The rapid increase in the production and global use of chemicals and their mixtures has raised concerns about their potential impact on human and environmental health. With advances in analytical techniques, in particular, high-resolution mass spectrometry (HRMS), thousands of compounds and transformation products with potential adverse effects can now be detected in environmental samples. However, identifying and prioritizing the toxicity drivers among these compounds remain a significant challenge. Effect-directed analysis (EDA) emerged as an important tool to address this challenge, combining biotesting, sample fractionation, and chemical analysis to unravel toxicity drivers in complex mixtures. Traditional EDA workflows are labor-intensive and time-consuming, hindering large-scale applications. The concept of high-throughput (HT) EDA has recently gained traction as a means of accelerating these workflows. Key features of HT-EDA include the combination of microfractionation and downscaled bioassays, automation of sample preparation and biotesting, and efficient data processing workflows supported by novel computational tools. In addition to microplate-based fractionation, high-performance thin-layer chromatography (HPTLC) offers an interesting alternative to HPLC in HT-EDA. This review provides an updated perspective on the state-of-the-art in HT-EDA, and novel methods/tools that can be incorporated into HT-EDA workflows. It also discusses recent studies on HT-EDA, HT bioassays, and computational prioritization tools, along with considerations regarding HPTLC. By identifying current gaps in HT-EDA and proposing new approaches to overcome them, this review aims to bring HT-EDA a step closer to monitoring applications.

**Graphical Abstract:**

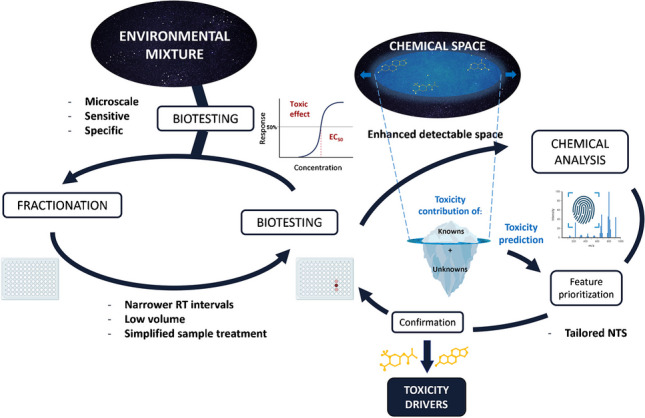

**Supplementary Information:**

The online version contains supplementary material available at 10.1007/s00216-024-05424-4.

## Introduction

Recent studies reported that over 350,000 chemicals and mixtures have been registered for production and global use [1]. Many of these compounds finally end up in the environment through different routes potentially having harmful consequences on both human and environmental health. With the advancements in the analytical field, in particular high-resolution mass spectrometry (HRMS), thousands of compounds and transformation products that might cause adverse effects can be detected in typical environmental samples. Although not all of these compounds contribute to the observed effects, the identification and prioritization of toxicity drivers remain extremely challenging [2]. Hence, novel and comprehensive approaches are necessary to find toxicity drivers for a reliable hazard and risk assessment. Effect-directed analysis (EDA) has emerged as an essential tool in addressing this challenge. This multidisciplinary approach combines biotesting, sample fractionation, and chemical analysis to unravel toxicity drivers in complex mixtures [3]. An EDA study begins by fractionating a complex sample, typically using chromatography, that has shown effects on the endpoint being tested. This process separates the sample into various fractions with eluting intervals of several minutes (usually between 2 and 3 min), containing fewer compounds and reduced matrix interference. The fractions are then subjected to biotesting to find those showing effects, or so-called active fractions, which will be then selected for chemical analysis to identify risk drivers.

The purpose of EDA can be divided into (i) identification/discovery of toxic compounds and (ii) quantification of the contribution of different risk drivers to the effects of complex mixtures. In early EDA studies, instrumental analytical capabilities restricted the identification of potential toxicants to either GC-(HR)MS/MS, LC–MS/MS, or LC-UV detector with limited possibilities of identification [4]. This changed drastically with the development of high-resolution mass spectrometry coupled to LC which allowed the detection and identification of previously unknown semi-/polar compounds.

Current monitoring applications generate extensive datasets with both analytical and bioanalytical tools. One of the main challenges for EDA is maintaining pace with these expansive screening datasets to pinpoint toxicity drivers. Moving from individual case studies to large-scale applications requires a leap in performance at every step of the process, improving traditional EDA workflows, which are generally labor-intensive and time-consuming, and do not guarantee successful identification of toxicity drivers. If we consider that the initial milestone in advancing the success of EDA was the integration of Suspect and Non-Target Screening (NTS) workflows, we currently witness the emergence of two additional ones: the implementation of high-throughput fractionation and biotesting, and the development of computational tools implemented in NTS workflows to enhance the overall success and speed of compound identification in EDA [3]. With these improved approaches, the so-called high-throughput (HT-) EDA aims to accelerate these workflows, relying on the following key features: (i) the combination of microfractionation and downscaled bioassays, (ii) the automation of sample preparation and biotesting to minimize manual intervention, and (iii) the use of tailored and efficient data processing workflows supported by novel computational tools to prioritize and identify the toxicity drivers.

The implementation of microplates (24-, 96-, or 384-well plates) in fractionation greatly reduces the manual intervention in intermediate steps such as sample transfer or evaporation of each fraction in separate vials or tubes [5]. This, in turn, significantly minimizes losses and contamination risks and improves the repeatability of the process. Additionally, the microplate format facilitates high-performance methods, allowing for almost simultaneous fractionation and biotesting of multiple samples, a concept that was previously unthinkable in traditional EDA using semi-preparative columns. This holds particularly true when making use of the latest advancements in instruments for automated in-plate fractionation, pipetting robots, or microplate evaporation systems. The toxicity of the fractionated sample can be tested easily with multiple endpoints, as substantiated by numerous recent studies [6, 7]. Since NTS remains one of the critical steps for HT-EDA, fast and effective data processing workflows for structure elucidation are required to efficiently explore the detectable chemical space, prioritize features with toxic potential, and pinpoint candidate structures that deserve further identification efforts. However, the fractionation and biotesting on microplates is not the only strategy that meets the demands of HT-EDA. Another viable approach is utilizing high-performance thin-layer chromatography (HPTLC) as an alternative to HPLC in EDA. The hyphenation of HPTLC and bioassays, called bioautography, enables the detection of toxicity drivers in complex environmental samples [8–10]. The direct accessibility of the analytes allows for the application of bioassays directly on the surface of the HPTLC plate resulting in an efficient workflow that produces effect profiles that can be directly used to compare samples along a temporal or spatial gradient or along a process such as wastewater treatment [11].

In our review, we provide an updated perspective on the state-of-the-art in EDA, high-throughput applications, and novel methods/tools that can be incorporated into HT-EDA workflows. Specifically, we have reviewed papers dealing with (HT-)EDA studies published since the last in-depth review in 2016 [3]. In addition to these, the search has been extended to include studies of particular interest on HT bioassays, NTS workflows, or computational prioritization tools with potential application in EDA. As an increasingly promising alternative to HPLC for HT-EDA, we also discuss specific considerations regarding HPTLC. Finally, based on the information gathered from the reviewed literature, we discuss the ongoing challenges that HT-EDA has yet to overcome and provide recommendations. It is important to note that this article does not intend to reiterate a comprehensive literature review of each individual step in the conventional EDA protocol (e.g., sampling or sample preparation), as this has been previously covered by other authors [3, 12]. Instead, it is the progress towards high-throughput applications that drives us to conduct this review and assess whether HT-EDA is indeed ready to go.

## Requirements, achievements, and challenges for bioanalytical tools in HT-EDA

Effect-based methods are bioanalytical tools that use the response of living organisms, cells, or molecular systems to detect and quantify the potency of chemicals and complex environmental samples affecting specific biological endpoints [13, 14]. With the overall aim of identifying toxicity drivers, effect-based methods serve as the primary tools to derive information about adverse effects caused by a mixture of compounds. Bioassays do not require a selection of known target compounds to be addressed but rather detect the biological activity of whole mixtures, considering all known and yet unknown compounds that interact with the bioassay [1]. In (HT-)EDA studies, bioanalytical tools are used in an initial step to identify bioactive samples and fractions, which will be further analyzed for toxicity driver identification. As a subsequent step, in the absence of toxicological data for the identified compounds, bioassays are performed to verify their activity by concentration–response relationships for the individual toxicity drivers and their mixtures. Through this approach, the contribution of these components to the overall detected toxicity of the fraction(s) and the sample itself can be confirmed and quantified.

HT bioassays can be defined as systems that allow testing of a large number of samples simultaneously or in rapid succession, efficiently and quickly. They usually involve automated procedures or robotics to achieve this goal. The utility of high-throughput bioassays for toxicity monitoring in large-scale environmental studies has already been demonstrated in the last years [14–16]. However, their application in HT-EDA studies demands even greater efficiency, as the activity of a substantial number of fractions, typically ranging from 60 to 300, must be measured in a time and cost-effective manner. Bioassays successfully used in HT-EDA meet some common criteria including miniaturization feasibility, high specificity, good reproducibility, automation capability, and high sensitivity. However, especially in the pursuit of such high performance, new approaches might bring some disadvantages and challenges that need improvement. Here, we discuss each of these criteria, reviewing the bioassays already implemented in HT-EDA, exploring novel potential approaches, and highlighting the challenges that lie ahead. The pros and cons of using in vitro vs. in vivo assays are also discussed under these criteria.

### Compatibility with HPLC-based HT-EDA

#### Scalability

For their compatibility with microfractionation (see the “Sample fractionation in HT-EDA” section), it is important that bioassays in HT-EDA are scalable to 96- or 384-well plates. This can facilitate the simultaneous testing of several samples with one or even multiple bioassays, as demonstrated in recent studies that exposed cells in parallel experiments to study at least three independent endpoints [7, 17, 18]. HT bioassays require a small sample volume as the test volume rarely exceeds 200 µL. This is a major advantage in extending HT-EDA to large-scale studies, as it avoids the need to handle excessive sample volumes. Most conventional EDA studies for water samples require sample volumes of tens to hundreds of liters, e.g., Hashmi et al. [19], Lopez-Herguedas et al. [20], and Massei et al. [21], which required 5, 25, and 850 L of water, respectively. In contrast in studies with HT microfractionation and specific in vitro bioassays, a grab sample of 100 mL of water [7], 150 mg of dust, or 9 mL of serum [22] may be sufficient, largely due to the reduced injection volumes achievable through microfractionation.

A wide range of in vitro bioassays in 96- or 384-well plate formats are described in the literature, giving them an advantage over in vivo bioassays that are often limited by the need for larger volumes [13]. These include several endocrine disrupting endpoints, mutagenicity, genotoxicity, cytotoxicity, aryl hydrocarbon receptor affinity, enzyme inhibition etc. [3]. In the literature, (anti-) androgenic, estrogenic, (anti-) progestogenic, glucocorticoid, mutagenic, and neurotoxic activities have been studied in HT-EDA workflows (Table SI.1) using 384-well plates following the (Anti-)AR-, ER-, (Anti-)PR- and GR-CALUX, Ames (*VM7Luc4E2*) and AchE inhibition assay protocols respectively [6, 7, 23]. In the same vein, microbial growth inhibition and transthyretin (TTR) binding assay in 96-well plate format also showed a good compatibility with HT-EDA [22, 24, 25]. Certain in vivo tests, however, have the potential to overcome the scalability issues and high volume requirements. For example, bioassays using *Daphnia magna* can be efficiently performed with comparable results to the classical approach, even in 24-, 48-, and 96-well plates by reducing the total volume and keeping a fixed surface to volume ratio [26]. Furthermore, modified versions of the FET can be conducted in 96-well plates, drastically reducing the final exposure volume (200 µL per well). Despite the potential of these bioassays, no studies that address their implementation for HT-EDA have been found. The only self-styled high-throughput in vivo bioassay used in the latest EDA works is a scaled-down version of the midge toxicity assay in 12-well plates [27, 28]. This assay was used to identify several toxic compounds and quantify their contribution to the toxicity of urban waterway sediment samples, showing a great potential for the assessment of contaminated sediment samples.

Beyond making the practical aspects of testing easier, microscaling also brings time and cost benefits. Most in vitro assays used in HT-EDA studies involve exposure periods of 24 h maximum (e.g., reporter gene assays such as CALUX or EcoScreen) or even 2-h exposure followed by 28-h incubation (Ames luminescence assay). Meanwhile, organismal assays typically have exposure times of 48, 72, or even 120 h, e.g., the *Daphnia magna* immobilization test, the midge toxicity test, and the algal growth inhibition test or the acute fish embryo toxicity test, which greatly lengthen the experiments [29, 30]. The cost savings are also related to the use of less material by unifying fractionation and biotesting in the same plate. However, the use of plates can also bring certain practical limitations, such as the impact of the materials used. In many in vitro bioassays, polystyrene well plates are used for cell exposure which might result in modification of the biological response due to leaching of, e.g., additives from the plastic or sorption of sample components to the plastic, depending on the physico-chemical characteristics of the contaminants in complex samples. This, for instance, has been shown by Johann et al. [31], where the estrogenic activity of oil-contaminated water-accommodated fractions in polystyrene well plates would have overestimated the real estrogenicity, likely due to leaching effects. While glass-coated alternatives are available, in which normal cell growth has been shown, these materials are more expensive, and not all well plate formats are available.

#### Specificity

Addressing bioassays with specific endpoints, such as receptor-based assays, significantly increases the chances of success in EDA since toxicity is caused by a few toxicity drivers. Furthermore, it increases throughput by facilitating their identification using endpoint-specific databases or toxicity prediction tools (see the “Identification of the toxicity drivers” section). In Table SI.1, some EDA studies using non-specific bioassays are shown, both in vivo and in vitro, such as the sea urchin embryo test or oxidative stress by ARE c32 [31–33]. Although these studies are more relevant to aquatic ecosystems, they also show a wider distribution of activity [21]. HT-EDA studies conducted thus far consider this aspect, with almost all of them employing specific in vitro bioassays, as mentioned earlier. Although in vitro assays provide valuable information, especially with their link to molecular initiating events (MIE) [34], in vivo bioassays provide higher ecological relevance. Consequently, the implementation of HT in vivo bioassays should be further explored in HT-EDA, despite the difficulties posed by their lower specificity. However, behavioral assays focusing on sublethal effects can increase the specificity of the test, allowing a higher chance of identification of compounds with specific MoA and can also be applied in low-volume plates in a high-throughput manner. In the last years, many studies have shown the application of behavioral assays with *Daphnia magna* in plates to screen the effect of neuroactive chemicals [35–37], and such tests could be applied also in HT-EDA studies for the identification of neurotoxicity drivers. Zebrafish (*Danio rerio*) embryo test (FET) represents also a promising tool since it allows the screening of a wide variety of endpoints, such as behavior, morphology, enzyme activity, and metabolomic patterns. A recent EDA study from Massei et al. [21] showed the potential of the acetyl cholinesterase (AChE) assay with zebrafish embryos for the identification of neuroactive chemicals in complex environmental mixtures. However, so far, the assay has not been miniaturized to be used in an HT-EDA study.

Modern techniques such as transcriptome analysis can also account for a holistic identification of toxicity pathways. In this approach, targeted RNA sequencing measures the expression of a selected set of key genes, typically around 1000–1500 [38]. For instance, Guo et al. [34] exposed a human permanent cell line (MCF7 cells) to water sample extracts, performed a reduced human transcriptome analysis, and identified some affected toxicity pathways, namely endocrine disruption (estrogenicity) and immune pathways. This identification was carried out using a virtual EDA approach where the sample effects were correlated with detected compounds by suspect and non-target screening without fractionation. The identified key drivers explained 54% of the estrogenic bioequivalent activity, which is in line with other EDA studies focusing on estrogenicity [20]. Although these high-throughput transcriptomics techniques have not been combined with fractionation to date, they align well with the requirements of HT-EDA and their joint potential is worth mentioning.

In addition, the implementation of biosensors as analytical tools has been recently proposed as complementary strategy to HT-EDA that will allow its application to on-site monitoring [39]. In this review, Li and Guo covered the latest developments on chemo/biosensors for toxicity testing and chemical analysis. These assays are based on a specific biological sensing element (DNA, protein, antibody, lectin, aptamers, or whole cell models) that produces a signalling element (i.e., fluorescence, luminescence, or color) when in contact with environmental pollutants present in a real-life sample or an extract [40]. However, these biosensors for the detection and semi-quantification of established biomarkers of toxicity still need further development, and this newly proposed SensorEDA needs to be tested with real-life samples.

#### Reproducibility

Reproducibility of the bioassay is another important aspect, as it allows throughput to be further increased by eliminating both technical and biological replicates. Though replication is an irreplaceable measure to cover biological variability, it might be sufficient to limit replication if the assay is used as a first screening tool in HT-EDA [17]. Hence, for exploratory identification of toxicity drivers, a single biotest may be sufficient to compare the toxicogram or bioassay chromatogram (i.e., the activity of the sample along the elution time) with the chromatogram, as demonstrated by Zwart et al. [17]. So far, most of the established HT-EDA workflows use a similar approach, where the number of biotests is reduced to a minimum. The contribution of the identified compounds is commonly calculated by comparing the EC_10/50_ of the compound and the full extract or fractions. If the unfractionated extract is used as the reference, testing of the dose–response curves of the fractions can be avoided [41, 42]. Although this greatly speeds up the process, there are a number of factors that can influence the results, even if the assay is highly reproducible. Firstly, the activity of the sample may be masked by the antagonistic effects of the mixture and fractionation can help to unmask them. For instance, Houtman et al. [6] found both progestogenic (PR) and anti-PR activities in WWTP influent samples, but their bidirectional masking was separated through fractionation. Building the dose–response curve of the fractions would help to quantitatively explain the contribution of the toxicity drivers on unmasked effects. In addition, the calculated concentration in the sample does not consider possible losses in the process and the matrix effect (i.e., ionization suppression or enhancement leading to under- or overestimation of the concentration) may also bias the results.

The testing of a recombined sample is also a common practice in conventional EDA [19, 20, 32, 43], which so far has been lost in HT-EDA studies. This reconstituted sample is used to assess the recovery of the toxicity by combining all fractions into a new sample, which is then tested under the same nominal concentration and conditions as the raw sample [3]. Pooling an aliquot from each fraction into a new well or vial could also work for QC in HT-EDA, although the maximum achievable REF would be limited. Therefore, even though these QA/QC practices work against high throughput, they are important criteria to be met for the design of fractionation protocols in HT-EDA [3]. Skipping the preparation of a recombined sample may be justified in scenarios where the sample volume is exceptionally restricted, such as in biological or human samples. As part of these good QA/QC practices, the evaluation of potential false positives should also be considered. For that, it is important to test procedural blanks alongside every sample. This allows the assessment and control of false positives due to interferences introduced either during the extraction process or during the chromatographic separation.

#### Automation

Another source of HT is automation, which is increasingly being integrated into bioanalytical work, for example using robotics for tasks such as applying exposure solutions via automated liquid handling robots [44]. This type of technology has only been applied in one HT-EDA work [24], although others have emphasized the need for it, especially if the workflow is to be extended to large-scale studies [7]. Technology in in vivo toxicity pipelines is also progressing towards high-throughput capabilities, including automatic pipetting, dechorionation and imaging [45, 46], contributing to bringing certain in vivo bioassays closer to HT-EDA. Although the authors describe the procedure as high-throughput, the midge toxicity test mentioned above did not make use of automated pipetting or image analysis-based counting technologies [27]. Furthermore, only four samples were studied in this study, so the potential of the bioassay in the context of HT-EDA has yet to be further demonstrated.

#### Sensitivity

Sensitivity of the bioassays is an even more critical aspect for a successful HT-EDA application. Overall, we observe a fine interplay between the number of fractions and the sensitivity of the bioassay that needs to be balanced in the HT-EDA study design. With increasing number of fractions, compounds are potentially eluted into several adjacent fractions, which reduces the concentration in one fraction and consequently the biological response. The influence of the number of fractions on toxicity can be deduced from three studies in which estrogenicity drivers in wastewater effluents were sought. Hashmi et al. [47] identified two active fractions occurring between minutes 14–16 and 28–30 (18 fractions in total), while Sonavane et al. [48] detected two active regions consisting of four and two active fractions, within minutes 39–51 and 54–60 (40 fractions of 3 min in total), respectively, both employing semi-preparative fractionation. In contrast, for the study with microfractionation by Zwart et al. [7], estrogenic activity was detected within minutes 11–14 and 15–16.5 with more than 10 fractions in each region (288 fractions in total), some identified compounds being the same in all three studies. In the first study, the contribution of the risk drivers could be successfully explained by constructing the dose–response curves of the fractions. In the latter case, even if a quantitative analysis of the contribution to toxicity was not sought, due to the large number of toxic fractions it would be infeasible to follow the same approach. Therefore, the alternative would be to use the raw sample as a reference, thus resulting in a biased explanation of the toxicity contribution(s) of the identified compounds. The width of the chromatographic peak limits the maximum resolution that can be obtained in the fractions, so increasing the number of fractions may not be beneficial beyond a certain number. The use of HT-EDA in this work undoubtedly reduced the workload, but decreasing the number of fractions could have facilitated the identification and calculation of the contribution.

The toxicity distribution does not only hinder the contribution quantification, but it can also lead to false negative responses and an underestimation of the hazard if the biological response falls below detection limits. To overcome this limitation, the concentration factor of the fractions could be increased. Sufficient sensitivity in the bioassay is also important to perform HT-EDA studies on some sample types where lower contaminant concentrations are expected, such as drinking water [49]. In that sense, most in vivo assays might not be sensitive enough and reporter gene assays would especially be recommendable due to their higher sensitivity. Depending on the potency of the substance, some reporter gene assays detect bioequivalents in low ng/L ranges [50]. In the analysis of surface waters, Zwart et al. [7] found an activity of 0.20 ng DHT eq/L and 0.05 ng E2 eq/L for AR-agonism and ER-agonism respectively.

### Compatibility with HPTLC-based HT-EDA

**Fig. 1:**
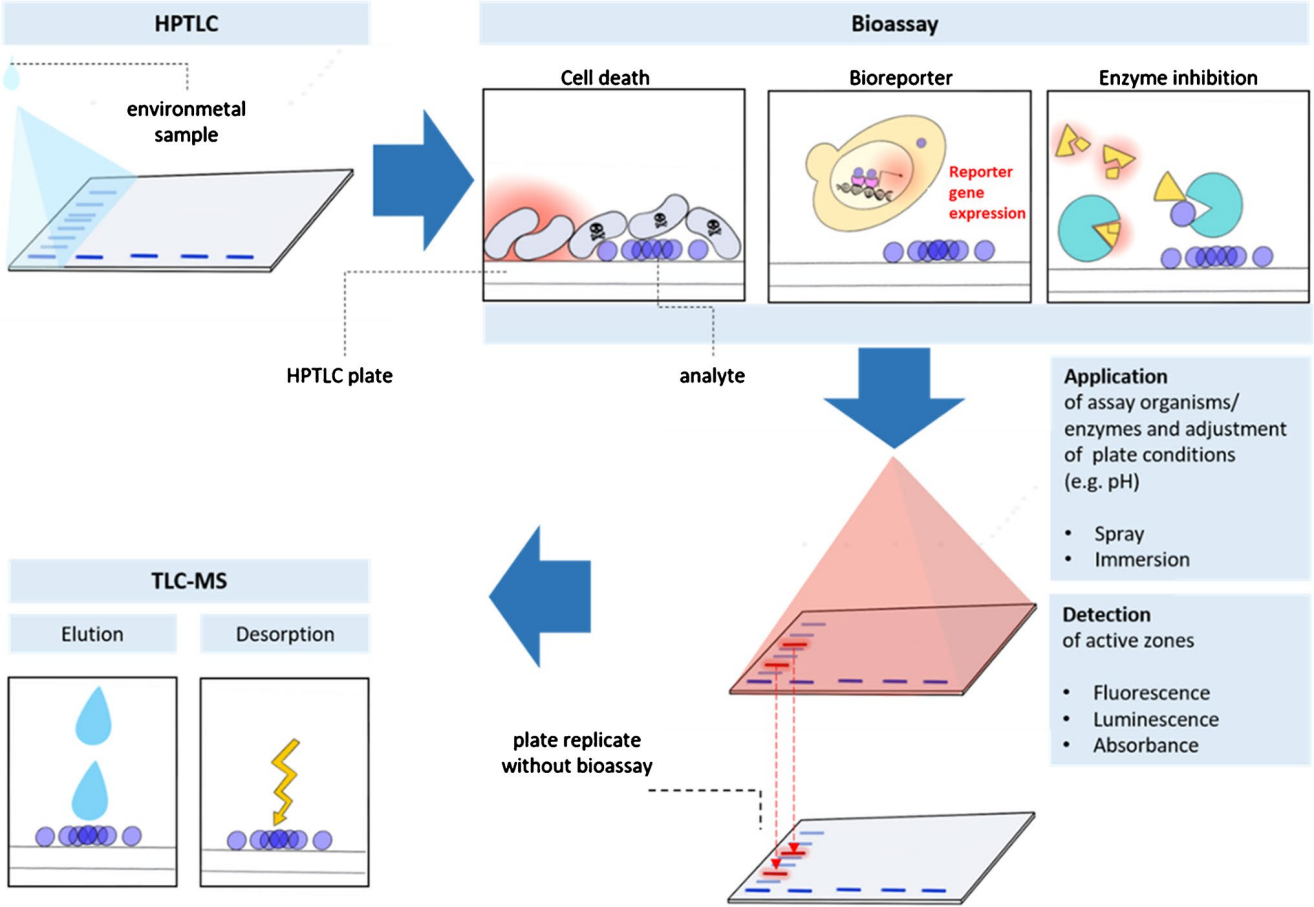
Bioautography workflow for HPTLC

Although the conventional strategy to address the requirements for HT bioassays involves the use of 96- or 384-well plates, HPTLC has also emerged as an attractive alternative. Despite having lower analyte separation efficiency compared to workflows based on HPLC (see the “GC-based separation” section), HPTLC undoubtedly provides high performance. This can greatly assist HT-EDA workflows that seek to identify potential toxicity drivers in an exploratory manner. Some of the bioassays we have already referred to can be implemented on the surface of TLC plates in a hyphenation technique called bioautography (Fig. 1). Briefly, after the application and separation of the sample on the HTPLC plate, a biological entity is applied directly on the surface of the HPTLC plate. In this way, the sample separation, toxicity assessment, and, even in certain cases, chemical analysis can be carried out on the plate. The miniaturization capacity is therefore also fulfilled in the HPTLC format, even more efficiently. Up to now, a diverse array of planar bioassays has been incorporated to HPTLC analysis (see Table SI.2), including enzyme activity-based assays, such as the inhibition of acetylcholinesterase [11]; biotests that measure physiological responses like the inhibition of bioluminescence and microbial growth [51, 52]; and even assays targeting specific endpoints such as the inhibition of photosystem II [53], genotoxicity [8, 54–56], or agonistic and antagonistic activation of (hormonal) nuclear receptors [53, 57, 58]. Although most HPTLC studies aim to identify toxicity drivers in a qualitative manner, the potential of this tool to quantify their contribution was also demonstrated by Stütz et al. [11], who explained a significant proportion of the neurotoxic effect by four main drivers. HPTLC technology has also advanced in the direction of high-throughput, especially with the development of multiple development step devices (see the “HPTLC-based separation” section) which, in addition to optimizing and accelerating the process, offer improved reproducibility [59]. Furthermore, a high sensitivity can be also achieved in HPTLC using specific reporter gene assays for detecting genotoxicity or cellular signal transduction mediated by nuclear receptors. Results from various studies even concluded that the p-YES was as or more sensitive than the classic L-YES [60–62]. Similar to the HT bioassays using 96- or 384-well plates, these tests also require low sample volumes and need short exposure periods. For example, *Alivibrio fischerii* luminescent bacteria can be used in direct contact with the HPTLC plate to measure metabolic activity and detect cytotoxic compounds in as short as 5 min of exposure.

The main practical challenge in HPTLC lies in adjusting the optimal conditions for bioautography, which primarily involves the compatibility of the stationary phase with the biological entity. For example, the pH of the plate can affect the cell viability, and the selection of the stationary phase can also impact the sensitivity of the assay since the uptake of analytes is determined by a partitioning between the surface of the plate and the cells. Further information on advances in stationary phase materials that are compatible with bioassays to improve separation can be found in the “GC-based separation” section.

## Sample fractionation in HT-EDA

The progress in high-throughput bioassays mentioned in the previous section has influenced the development of new fractionation technologies. For instance, to maintain an automated workflow, microplate fractionation instruments coupled to HPLC have emerged, substantially enhancing the efficiency of EDA. Furthermore, separation in HPTLC has also significantly improved, and combined with the capability of applying certain bioassays on the same plate, has resulted in increasingly efficient EDA studies. In both cases, these improvements not only speed up EDA protocols and bring them closer to monitoring demands, but the narrower fractions also allow a reduction in the number of candidates to be identified, increasing the chances of success. The following subsections will cover the advances in fractionation/separation using HPLC and HPTLC implemented in HT-EDA and discuss the challenges and remaining needs. Despite its low representation in the literature, fractionation after GC will be briefly discussed as well.

### HPLC-based HT microfractionation

HT microfractionation presents an alternative to traditional fractionation within EDA which typically involved 15–30 fractions, each lasting 1–3 min. Instead of semi-preparative columns that require higher flow rates and thus large volumes, microfractionation uses analytical columns that provide improved chromatographic resolution, as well as a larger number of fractions in reduced volumes, thereby shortening retention time intervals (60–300 fractions, each lasting 6–30 s). In HT-EDA, the same analytical column and chromatographic conditions can be used for fractionation and HPLC-HRMS analysis, allowing a more straightforward comparison between the MS chromatogram and the toxicogram without the need for up- or down-scaling calculation and the associated errors, e.g., retention time shifts and peak shape differences [25]. One of the main advantages of this approach is that it simplifies EDA workflows notably when the aim is to discover toxicants rather than to quantify contributions. For this aim, an initial screening of the toxicity of the fractions with the appropriate enrichment factor would be sufficient (“Requirements, achievements, and challenges for bioanalytical tools in HT-EDA” section) and it may not be necessary to perform additional chemical analyses on the fractions. Microfractionation also allows for smaller volume fractions, leading to reduced injection volumes and sample consumption. This is another key reason why smaller sample volume is required in HT-EDA, making it more suitable for monitoring studies.

In addition, by increasing the separation power due to having shorter fractions, this approach also allows a better differentiation between endogenous molecules that might pose an intrinsic effect on the bioassay, and potential environmental pollutants with similar elution patterns. This is of great importance when HT-EDA wants to be performed in samples with a more complex matrix, such as wastewater influent, biota, or human samples. Fractionating by analytical columns also reduces the solvent consumption due to lower mobile phase flow rates. General recommendations for chromatographic column choice and separation methods (i.e., stationary phase chemistry, column dimensions, gradient condition, etc.) in HT fractionation align with those for any NTS workflow dedicated to environmental sample analysis [63]. The objective is to attain optimal separation performance for a broad spectrum of compounds while maintaining short programs for time and cost efficiency, making C18 columns the most prevalent choice in HT-EDA (see Table SI.1). When investigating highly hydrophilic compounds, it may be worth considering alternatives such as hydrophilic interaction liquid chromatography (HILIC) or mixed mode LC (MMLC) columns, although these have not yet been investigated in this particular context.

This high resolution with smaller intervals per fraction requires cutting-edge technology capable of rapidly and accurately collecting the fractions [5, 25, 64]. Novel solenoid valve-based fractionation systems, such as the Fractiomate™ [5], enable the accurate spotting of the sample on microplates on a base that moves along the x–y axis, allowing the distribution of fractions according to user preferences. The miniaturization of this key step has allowed EDA protocols to fractionate multiple samples on the same day compared to the time-consuming multi-day processes by semi-preparative fractionation. This is particularly useful in combination with highly sensitive small-scale in vitro bioassays (e.g., hormone receptor activation assays) (see “Requirements, achievements, and challenges for bioanalytical tools in HT-EDA” section). This approach allows the desired enrichment factors to be achieved with a single injection, and the well plates can then be evaporated and reconstituted into the bioassay medium for rapid testing. Zwart et al. [17] used a single injection for parallel toxicity testing and chemical analysis by splitting the column flow between the fraction collector and the MS. Yet, a limitation of microfractionation systems like the Fractiomate™ is their current inability to facilitate the collection of the recombined sample for quality control purposes, as they are exclusively designed for microplate fractionation.

**Fig. 2:**
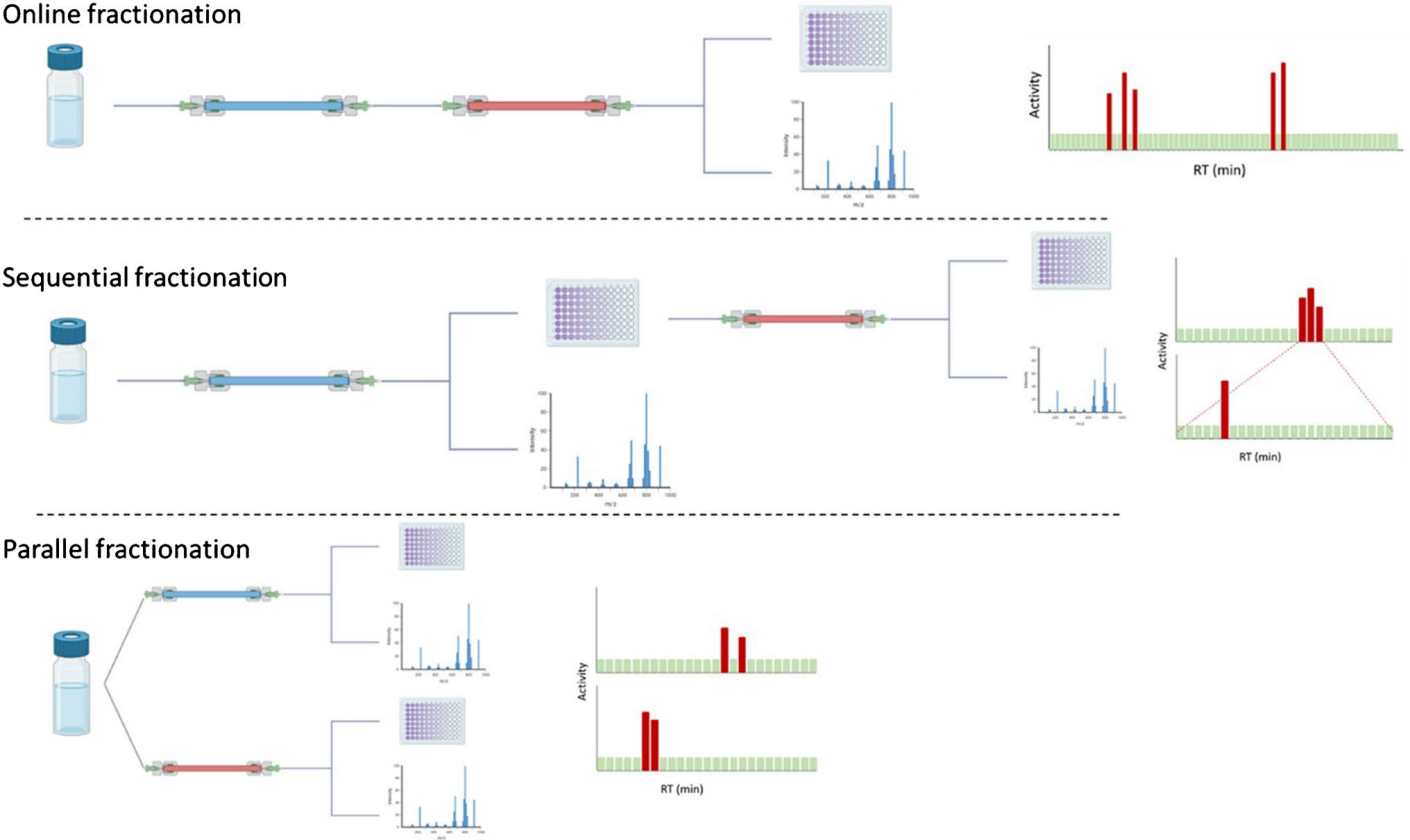
Overview of the different orthogonal fractionation strategies

In conventional EDA, the use of orthogonal fractionation is a commonly used strategy to improve the chances of success with highly complex samples. In this strategy, the analytes are separated by two or more successive or parallel chromatographic columns with highly distinct selectivity, thus bringing additional dimension(s) to the separation (Fig. 2). Although it is a priori opposed to high throughput, its potential to facilitate the identification of toxic compounds should be also considered in HT-EDA. In the typical scenario, the toxic fraction undergoes a second fractionation to further reduce the number of compounds in the fractions. The identification of risk drivers is then focused on a reduced list of compounds detected after the second step, and further filtered by keeping only those detected compounds common to both steps. However, implementing this method, known as sequential fractionation, in HT-EDA with microplate fractionation poses challenges due to the increased difficulty in achieving the necessary enrichment factor for the second step. There are two orthogonal fractionation alternatives that would be more compatible with HT-EDA. The first is parallel fractionation, where the raw sample is fractionated on two columns providing orthogonal separation and compound identification is focused on common compounds from the toxic fractions of both methods. However, this alternative does not allow a second simplification of the toxic fractionation and duplicates the bioassay and fractionation work. The second alternative, and the only one explored so far in HT-EDA, is online fractionation. In this approach the fractions are collected after the samples pass through two online orthogonal columns and fractions are tested only once. In Ouyang et al. [65], an LC x LC separation was achieved using C18 and PFP columns and fractions collected in 384-well plates. The orthogonality of this method yielded a high chromatographic resolution, coupled with HT fractionation, leading to the identification of tiapride, amisulpride, and lamotrigine, which entirely explained the observed effects of 3 out of 7 fractions (Table SI.1). Although this method fits well with HT-EDA and greatly simplifies the matrix, it does not allow cross-checking of common compounds from sequential or parallel toxic fractions to filter compounds as deeply. In general, as with quality control practices, these strategies can be explored in HT-EDA, even if they reduce performance, as they help to make HT-EDA more successful.

### HPTLC-based separation

Thus far, our emphasis has been on studies employing LC/HPLC for chromatographic separation coupled with post-column fractionation. In contrast, HPTLC offers separation on a two-dimensional surface. Compared to the classical TLC, HPTLC plates have lower diffusion of analytes due to the significantly smaller particle size of the novel plate materials, which results in enhanced detection sensitivity and analysis speed. Furthermore, this improvement facilitates more efficient application of bioassays, identification of narrower toxic regions or spots, and direct elution or desorption of analytes, which makes HPTLC a robust choice for HT-EDA. By incorporating all these steps on the plate, efficient HT-EDA workflows that should be considered for large-scale monitoring can be reached, especially when the goal is solely the discovery of toxic compounds. When appropriate chromatographic conditions are combined with compatible bioassays, the use of HPTLC can even transition from the qualitative screening to identify potential toxic compounds to the quantitative analysis of their contribution to the total toxicity of the sample [7]. Moreover, technological advancements are aiding in enhancing peak capacity in HPTLC, reducing the resolution gap compared to HPLC. As recapped by Weiss et al. [59], the commercially available product range for HPTLC hardware/chambers had been extended by multiple development step devices (automated multiple development, AMD), performing several consecutive plate developments with changing mobile phases [59]. The main disadvantage of this method is that it is more time-consuming than single step development. In addition, the improved peak capacity is wasted if the bioassay used increases the diffusion of the analytes, which is often the case for bioassays with long exposure times. Thus, bioassays with a short exposure time, such as the bioluminescent bacteria test, can be usefully combined with AMD, whereas other assays, such as the pYAAS, might need fixation of the compounds before testing [66].

Currently, stationary phases consisting of spherical silica particles, as already established for HPLC approaches, are available, promising enhanced performance. In comparison to classic TLC silica plates, an improved separation could be demonstrated for hormones (estrone, 17β-estradiol, 5α-dihydrotestosterone, progesterone) on these plates [57] and they had successfully been used in acetylcholinesterase inhibition bioautography experiments [11, 67]. While the first results on the separation performance of plates with spherical silica particles seem promising, their influence on bioassay sensitivity still needs to be investigated in more detail since a loss in sensitivity was indicated [68]. This could be due to the altered structure of the stationary phase hampering the interaction of microbial test organism with the surface of the HPTLC plate. As an alternative to the classic normal phase separation using a silica matrix, C18-modified reversed phase (RP) plates might be used. The big advantage of non-polar stationary phases is that due to the weak elution strength of water, the performance of the aqueous bioassay on the plate surface does not increase the diffusion of the analytes. For example, Klingelhöfer et al. [52] developed a novel method to detect (anti-)androgenic compounds in cosmetics and thermal paper using RP-18W plates. Another aspect to consider is the pH of RP HPTLC plates which is comparatively low (~ 4.7) [69]. This means that preconditioning of the plate is necessary before applying the assay organisms, as shown for the bioluminescence inhibition assay (*Aliivibrio fischeri*), p-YES or p-YAS [52, 68, 69].

For reliable identification of compounds within the active zone (i.e., the spots of the HPTLC plate where activity is found), elution-based methods are the most common approach for transferring compounds from HPTLC plates to MS [11]. Other transfer methods belong to the desorption-based approaches like MALDI etc. and are reviewed in detail elsewhere [70, 71]. The coupling of HPLC to a subsequent MS analysis is technically much more advanced compared to the MS analysis of HPTLC plates. In this way, the combination of HPTLC with HPLC-HRMS is the best approach when seeking an in-depth EDA. EDA workflows, including 2D-HPTLC followed by HPLC-HRMS, have been used, for instance, for the identification of neurotoxic substances (acetylcholinesterase inhibition assay) in water samples [11].

Regarding orthogonal separation in HPTLC, two-dimensional coupling of HPTLC developments has also been performed. Initially, the sample is first chromatographically separated along the first axis. In a consecutive separation step with another mobile phase, the analytes are afterward separated along the perpendicular axis, resulting in the orthogonal separation of analytes throughout a two-dimensional plane. In contrast to orthogonal separation in HPLC, where different columns are needed, here two-dimensional chromatography on one [72] or two HPTLC plates [11, 73] can be adapted to EDA workflows. However, both come with disadvantages as in the first case only one sample can be tested per plate while the second case involves an additional sample transfer. After the 2D separation, the number of candidate compounds can be greatly reduced, as demonstrated by Stütz et al. [11].

All in all, HPTLC-based approaches for EDA can be established as high-throughput approaches to be used mainly for the exploratory identification of toxicity drivers while they should be further developed for more quantitative analysis of toxicity contribution. It remains to be demonstrated that these methods work on a larger scale, as has been shown so far only in areas such as the routine detection of adulterants in milk [74]. Details of recent EDA studies using HPTLC, including endpoints, sample type, detection method, stationary phase and mobile phase, are given in Table SI.2.

### GC-based separation

Despite the obvious challenges associated with using gas chromatography for the separation and subsequent collection of fractions, this method was frequently employed in the past, when the focus was primarily on (semi-)volatile compounds and LC-HRMS was not available. The main methods for collecting fractions included using cold or adsorbent traps [75] but more modern techniques implemented the use of solvent infusion after GC column [64, 76]. The latter was used by Pieke et al. [76] to collect the column outcome via a condensation capillary directly into 96-well plates, allowing a HT microfractionation that was the precursor of the Fractiomate™ (see the “HPLC-based HT microfractionation” section). This type of technology can be coupled to general purpose non-polar columns to cover a wide range of chemicals which in turn are recommended for NTS [63]. The main challenge of this strategy is the potential loss of the more volatile compounds in the fractionation and evaporation stages. For chemical analysis, this can be avoided by separating the effluent from the column to the MS detector and the fraction collector, as demonstrated in the improvement of the above system by Jonker et al. [64]. However, for toxicity analysis, evaporation is mandatory and avoiding these losses is difficult. From 2015 to the date of this review, the literature on this particular method has been very limited, with only one article reporting the application of GC-based EDA to real samples [77]. In that work, authors studied the potential effects of AhR active compounds adsorbed on microplastics deployed in the Great Barrier Reef, Australia by EDA. Although an adaptation of the HT microfractionation system mentioned above was used, the fractions were collected in vials, decreasing performance. The results of this study suggest that the recoveries from this process should be further investigated and optimized.

## Identification of the toxicity drivers

From data acquisition until the last step of structure elucidation of risk drivers, HT-EDA data processing workflows must comply with the requirements of high-throughput while maintaining or even improving the likelihood of success. If the advances outlined in the previous sections have ensured that an increasing number of samples can be fractionated and tested in a short timeframe, the identification of toxicity drivers must keep pace and not be the bottleneck. The main criterion to make an NTS high throughput is effective feature and candidate structure prioritization. Unlike NTS workflows for other purposes, in (HT-)EDA most of the features detected are irrelevant to the end goal, i.e., prioritization of toxicity drivers is key. An inappropriate prioritization strategy, among others due to a feature list derived from an analytical method lacking coverage of the right chemical space (“Instrumental analysis in HT-EDA” section) or due to raw MS data failing to meet quality standards (“Suspect and non-target screening (NTS) workflows in HT-EDA” section), may in turn lead to toxicity drivers being overlooked. Chemical analysis and compound identification efforts must find a balance between covering the largest detectable chemical space while filtering effectively to isolate the smallest number of potentially toxic compounds. The following subsections delve into the analytical methods and NTS workflows employed in HT-EDA studies. The discussion covers not only potential advancements to enhance performance but also to ensure the success of the studies. Finally, we review the computational tools based on machine learning algorithms for toxicity predictions which hold significant potential in the future of HT-EDA.

### Instrumental analysis in HT-EDA

Identification of active compounds in HT-EDA studies mainly relies on liquid chromatography-mass spectrometry (LC–MS). In fact, HPLC-HRMS was used to identify toxic compounds in all the studies reviewed that used microfractionation. All but two were carried out on aqueous samples, so the range of compounds expected is suited to this technique. In one of the exceptions, microfractionation was used to analyze sediment samples with a focus on PFAS, but HPLC-HRMS is typically preferred for the analysis of this family of compounds also in non-aqueous matrices [78]. Common to almost all HT-EDA studies is the use of electrospray ionization (ESI) as the ionization source. ESI has the broadest coverage of the chemical space compared to other soft ionization techniques, such as atmospheric pressure chemical ionization (APCI) and atmospheric pressure photoionization (APPI), covering polar to semi-polar compounds. For this reason, ESI is often the first choice for the analysis of organic contaminants in water. However, studies showed the benefit of using multiple ionization modes (mainly ESI and APCI) to increase the coverage of compound classes [79–81]. One HT-EDA study used complementary ESI and APCI to analyze estrogenic compounds in consumer electronics plastics. In this study, BPA, a BPA-analogue, and 2,4-di-tert-butylphenol were identified as the main potential toxicity drivers [82]. However, all were detected in both ionization modes, so APCI was used as a second confirmation of identification rather than to detect new compounds. Because of the duplication of effort in data evaluation, it is more advisable to use complementary ionization techniques in a stepwise fashion, unless the objective of the study requires otherwise. For instance, if the aim is to quantify contributions and this is not accomplished using compounds identified by ESI, attempting APCI may be recommended.

GC–MS analysis has been most commonly used in conventional EDA studies for sediment samples and for endpoints such as aryl hydrocarbon receptor activity, where the expected active compounds are semi-volatile (Table SI.1). In addition to the type of samples investigated so far in HT-EDA, another reason why GC–MS analysis has not yet been implemented is the significant gap in NTS workflows compared to LC. In GC–MS, electron impact (EI) ionization (typically 70 eV) is the most common technique for non-polar chemicals, where the ion form of the intact molecule is rarely preserved. This brings challenges to the identification of certain classes of compounds where the MS and the MS/MS spectra are unspecific. Soft ionization can also be achieved in GC–MS by using low-energy EI, APCI or APPI as the ionization source. Since the peak of the molecular ion will be preserved, NTS workflows developed in LC-HRMS can be adapted for GC-APCI-HRMS data. Additionally, the GC retention index library can also be used with APCI measurements, which brings further confidence in the identification [83]. GC-APCI-HRMS might therefore be a potential tool to consider in the design of HT-EDA workflows for the identification of novel semi-volatile hydrophobic contaminants in sediments and other environmental matrices beyond water.

Similarly, ion mobility spectrometry (IMS) is another state-of-the-art technique that has not yet been investigated in HT-EDA but has significant potential to assist in the identification and prioritization of compounds in NTS. Briefly, in IMS, ions are subjected to an applied electric field and experience collisions with the gas molecules, leading to a change of velocity influenced by their size, shape, and charge. Therefore, an additional dimension of separation is added to chromatography, MS, and MS/MS for compound identification. IMS has the ability to filter interferences if co-eluting compounds have different drift times in the IM dimension. These drift times can be converted into cross collision sections (CCS) for each compound, a value that is independent of chromatographic conditions and unaffected by matrix effects. CCS can be used in compound identification as an additional physico-chemical property to aid identification in a similar way to retention time. This was demonstrated in studies by Menger et al. [84] for biota samples and by Celma et al. [85] for environmental water samples were data-independent data acquisition approaches were used.

### Suspect and non-target screening (NTS) workflows in HT-EDA

Environmental monitoring efforts have increasingly shifted from the analysis of a targeted, yet limited, set of known contaminants towards the use of broad screening methods allowing the simultaneous detection of hundreds to thousands of signals in a single analysis [14]. This shift has been facilitated by advancements in high-resolution mass spectrometry (HRMS) and associated NTS workflows. Nonetheless, it has been estimated that less than 5% of the NTS features measured in environmental and biological samples are commonly identified by a combination of target, suspect, and non-target in silico identification efforts [86]. Effective prioritization strategies are essential due to the large number of features detected and the extensive efforts required to elucidate their structure [63]. Moreover, depending on the context and research question, not all detected features hold the same significance [87]. In this regard, (HT-)EDA plays a crucial role by directing the identification only towards compounds eliciting relevant biological effects [25, 88]. Although substantially reduced, the number of features per fraction requiring investigation to explain observed effects can still reach several hundred or more, depending on the type of matrix, considered endpoint, and total number of active fractions [33, 41, 89]. Therefore, if a feature is not prioritized, or identified, we are blind to its toxic potential in (HT-)EDA, which can partially explain why only a small part of overall mixture toxicity is sometimes elucidated [90]. Therefore, workflows used to process NTS data for (HT-)EDA applications must be optimized to facilitate the prioritization and eventual identification of relevant features in active fractions.

After the acquisition, data from (HT-)EDA studies typically undergo (pre-)processing through workflows involving multiple steps also used in conventional NTS applications (e.g., data conversion, centroiding, compression, feature detection, componentization, and alignment over samples) [25]. Outputs, which are generally presented in the form of feature tables, require further verification using a combination of QA/QC, internal standards (IS), and blank samples. However, in (HT-)EDA applications, the use of IS is often limited given that these compounds might also induce effects in the bioassays [91], unless two different aliquots (with and without IS) are fractionated for chemical analysis and biotesting, respectively [19]. Furthermore, care must be taken to ensure that bioassay and MS chromatogram profiles are properly aligned to minimize errors in associating features to specific fractions [25]. Recently, several approaches have been proposed to facilitate and expedite the annotation, prioritization, and eventual identification of features detected in EDA studies. For example, employing specific suspect lists and spectral libraries containing information about compounds known to be (potentially) active on specific endpoints could help diminish the number of false positives and enable the elucidation of relevant suspected compounds [42, 92]. The NORMAN suspect list exchange (NORMAN SLE) currently comprises 111 suspect lists [93]. These lists include various endpoint-specific suspect lists, such as human neurotoxins, algal, and phytotoxins [94–96]. While suspect lists are a valuable tool for identifying and annotating known contaminants, they do not contain all compounds potentially present, and therefore, the risk of overlooking potentially relevant chemicals remains. This can hinder the discovery of new biologically active chemicals.

To overcome these limitations and allow for the detection and identification of novel unknown chemicals, several approaches have been proposed to tackle the large number of features present when working in non-targeted mode. These strategies can be divided into those that facilitate structure elucidation and annotation of long feature lists, and those that prioritize and filter both feature lists and candidate structures for each feature, according to the objective of the study. While some of them have already been employed in HT-EDA, others with significant potential remain unexplored and will be discussed here as well. A commonly employed method in NTS for identifying the structure of features of interest in the absence of matches with spectral libraries or mass lists is the use of in silico prediction tools, such as MetFrag [97], SIRIUS [98], or CFM-ID [99]. While the exact principles for the generation of the predicted spectra vary depending on the technique (rule-based, machine learning-based, or combinatorial), their general operational principle is the same: to retrieve MS and MS/MS data, predict the molecular formula, and tentatively identify the molecular structure by comparing the experimental and the in silico generated molecular fingerprints or fragmentation patterns based on entries in chemical repositories. For instance, MetFrag was implemented in the HT-EDA data processing workflow by Jonkers et al., [25] to enhance the high-throughput identification of contaminants.

The structural elucidation of features mentioned above is usually applied to feature lists that have been previously filtered using the prioritization tools that fit the objective of the study. Furthermore, as the number of potential candidates for each feature may vary, some tools can also be used to filter between candidates. In the same example as above by Jonkers et al., and others in (HT-)EDA [7, 32] retention time prediction was used as a post-processing tool to further reduce the number of possible candidate structures for prioritized features. Another MS2-based approach that could be particularly useful for the processing of HT-EDA/NTS data are so-called molecular networks [100]. They allow for the visualization of related features based on similarities in their MS2 spectra and are one of the main data analysis approaches used in the Global Natural Products Social Molecular Networking (GNPS) database [101]. Molecular networks can aid in the prioritization and identification of unknown potentially bioactive compounds in HT-EDA by visualizing their structural similarities with other known chemicals present in the same sample or fraction. Similar to RT, CCS prediction tools can also help to narrow down the list of candidates in each feature when IMS is used. This was demonstrated in the study by [102], where it was shown that the CCS prediction error was less than 6% for 95% of the compounds. Several machine learning prediction tools have emerged for that purpose [102–104]. Alternatively, an interesting approach for feature prioritization involves the use of multivariate statistics, such as partial least squares (PLS), which is occasionally referred to as virtual (v)EDA [105]. These methods prioritize features of interest by detecting particular patterns (e.g., temporal or spatial) within (unfractionated) bioassay data and linking them to analogous patterns in feature intensities [106, 107]. While these approaches require adequate and representative sampling, as well as sufficient variability in activity between samples to derive significant statistics, they are generally applied in an untargeted manner, allowing the prioritization of previously unknown features. In the large-scale scenarios targeted by HT-EDA, meeting this requirement should not be an issue so this strategy could prove especially beneficial. To further reduce the number of features in EDA/NTS, prioritization based on the presence of specific fragment ions can be an effective approach. For example, Loewenthal et al. [108] recently showcased how organophosphorus acetylcholinesterase inhibitors could be prioritized in HT-EDA by identifying indicative fragmentation ions. Prioritization techniques based on toxicity prediction from candidate structures or fragmentation spectra are covered in detail in the “Supporting tools for toxicity driver prioritization” section.

The efficiency of some of the approaches mentioned above strongly depends on the quality of MS2 data used as input to predict structures, perform library searches, or visualize structurally related compounds. Intelligent MS2 acquisition workflows have been recently developed by vendors precisely to enhance the spectrum quality, such as AcquireX in orbitrap systems from Thermo and Iterative MS2 on Agilent. In these workflows, a first full MS analysis is used to build the m/z inclusion list and exclusion lists from the sample and blank, respectively. These lists are then used in a second run to trigger the MS2 acquisition in the data-dependent-MS2 acquisition. Post-acquisition, triggered features are moved to the exclusion list, and the process is iteratively and automatically repeated until the MS2 acquisition of all features in the sample is achieved [63]. In addition, recently developed machine learning-based models to automatically assess the quality of MS2 spectra [109] could further improve the performances of the abovementioned tools for both prioritization and identification of relevant features detected in an HT-EDA study. Models that allow for the semi-quantification of suspects [110, 111] or even unknowns (based on their MS2 spectrum) [112] based on ionization efficiency and without the need for corresponding reference standards have gained significant attention in the NTS community. Although their application in the field of HT-EDA has not yet been thoroughly explored, semi-quantification approaches could be particularly useful to prioritize features based on risk [113] or to estimate their contribution to the observed effect. Semi-quantification tools could be of particular interest in HT-EDA studies that aim to quantify the individual contributions to the sample toxicity. Until now, these studies could only consider compounds for which a reference standard was available, which severely limited the possibilities. The introduction of these tools, and in particular the toxicity prediction tools listed in the following section, could completely change the HT-EDA landscape and also bring the unknowns into the contribution calculations.

### Supporting tools for toxicity driver prioritization

The aforementioned prioritization strategies based on analytical and statistical approaches help us to reduce the list of features for identification efforts, but they do not provide information about the features’ toxicological importance. Computational toxicology methods are based on the observation that resembling chemicals often have similar properties and cause analogous toxic effects. We can thus use machine learning to teach algorithms to entangle complex patterns in structural data leading to toxic behavior and use the developed models to predict the toxicity of new compounds. These approaches can be used to prioritize candidates in (HT-)EDA studies where several candidates are detected in the toxic fractions. Examples of utilization of various in silico tools to support feature prioritization in the context of EDA include the works of Gwak et al. and Cha et al., [18, 114, 115]. These studies utilized QSAR-based toxicity prediction to prioritize the elucidated candidate structures. In the former case, VirtualToxLab [116] and VEGA QSAR [117] were used to confirm the binding affinity of the candidates with AhR. Similarly, Cha et al. [114, 115] extended the endpoints addressed to confirm AhR, ER, and GR binding affinity using the same tools. Besides these two tools, both in vivo and in vitro toxicity data have been used as target data for the modelling of a wide range of tools yet unexplored in (HT-)EDA. From the environmental point of view, aquatic toxicity [118, 119], mutagenicity [120], and the Tox21 data endpoints [121], in particular endpoints expressing endocrine disruption [122–124] are the most commonly predicted toxic outcomes. MLTox [118, 125], deepFPlearn [126, 127], and TrendProbe [128] are of particular interest and hold significant potential for HT-EDA implementation as they were developed for environmental applications.

The structural input to train these models is often obtained from common databases such as CompTox Chemistry Dashboard [129]. However, these structures are not used directly as input but; instead, after rigorous cleanup [130, 131], they are converted into mathematical representations of molecules as fingerprints (binary strings of 0/1 s-containing bits indicating a particular substructure’s presence or absence) or molecular descriptors [132]. This also brings with it a major challenge, which is the high dimensionality of the input features (i.e., number of bits in molecular fingerprints, often several thousand) [133–135] that can slow down the data evaluation. Another challenge is that not all models are trained covering an adequate distribution of the chemical applicability domain. Some are trained on a specific chemical space and are used as universal conveyor belts for predictions of a broad spectrum of chemical structures, leading to biased results. Beyond these technical challenges, another drawback of this approach for implementation in HT-EDA is that they require an elucidated structure, so they can only be applied towards the end of the NTS to prioritize candidates within each feature.

**Fig. 3:**
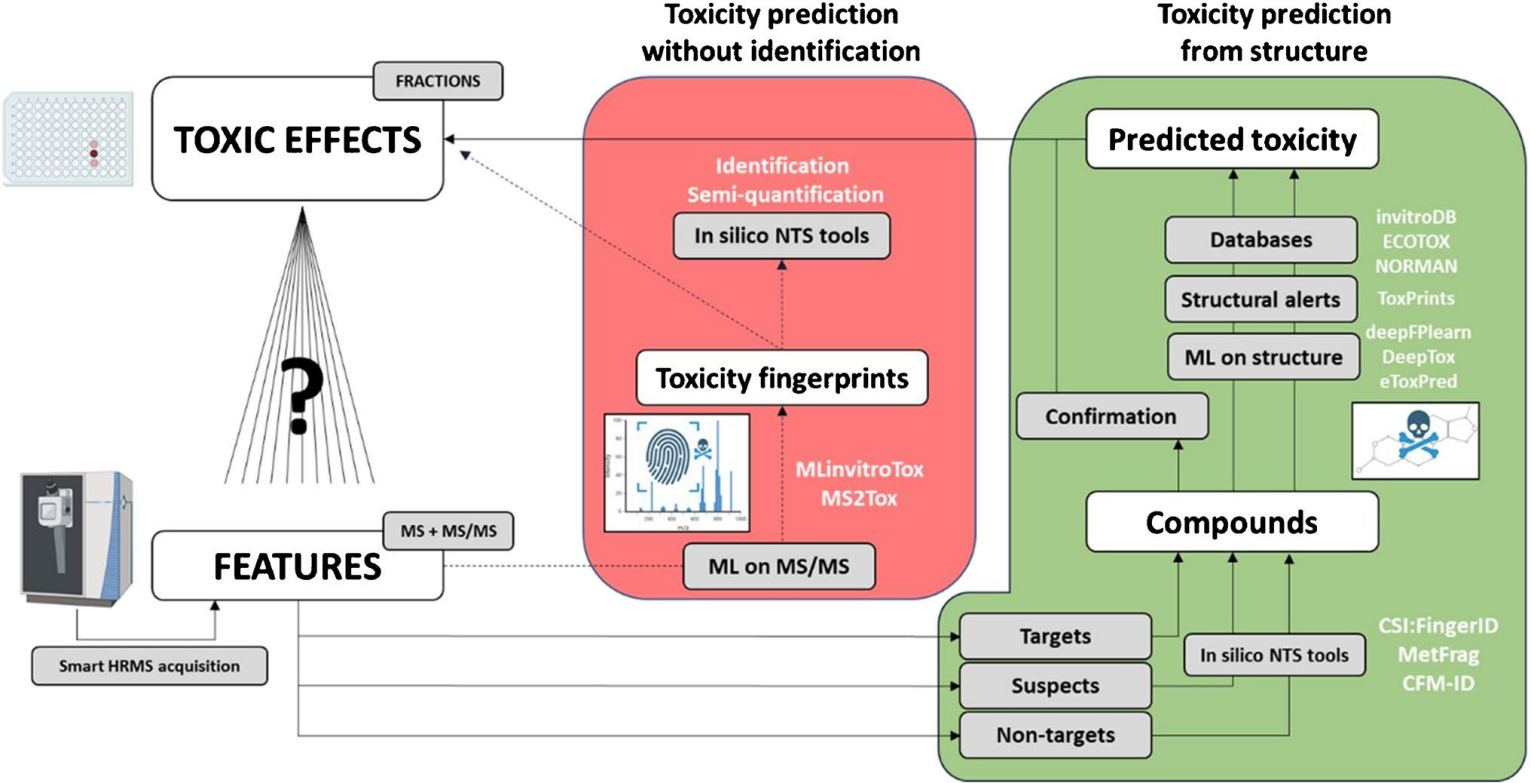
Landscape of toxicity driver prioritization and identification. The green zone shows the traditional analytical path via identification, toxicity assessment, and mapping to the original effects. The red zone shows the recently developed approach prioritizing the toxicity drivers via ML models based on the MS/MS spectra of the measured features, followed by the identification of the relevant candidates

Thus, an additional shortcut that can filter these extensive feature lists is required to address the implementation of HT-EDA in large-scale scenarios. The models mentioned above require the structure to generate these fingerprints, which are then used to predict toxicity. However, this is not the only way to obtain these fingerprints. Recent advances such as CSI:FingerID (integrated into SIRIUS) [98] allow molecular fingerprints to be generated from fractionation spectra. On that basis, two corresponding approaches were proposed in parallel by Peets et al. (MS2Tox) [136] and Arturi and Hollender (MLinvitroTox) [137] for predicting the toxicity of unidentified features based on the molecular fingerprints derived from MS/MS spectra instead of structures. As elucidation of unknown compounds’ structure is still the major bottleneck of all approaches utilizing in silico annotation tools, circumventing this step is expected to significantly streamline the data processing (Fig. 3). Exactly for this reason, this strategy aligns well with what is sought in HT-EDA, as it prevents wasting efforts on elucidating structures of features that are not relevant to the endpoint of interest. MLinvitroTox used in vitro HTS ToxCast and Tox21 data for training of supervised classifiers predicting a 1/0 binary hit call (toxic/nontoxic) for 500 + endpoints covering a broad spectrum of molecular toxicity endpoints such as AR, ER, PR (androgen, estrogen, and progesterone endocrine receptors, respectively), AChR (acetylcholine receptor and neurotransmitter), p53 (tumor suppressor), AhR (aryl hydrocarbon receptor responsible for cell metabolism), OSR (oxidative stress response), and IR (inflammatory response). This tool covers most of the endpoints addressed so far in HT-EDA studies. MS2Tox used also in vivo aquatic toxicity data and is able to predict LC50 of chemicals in, e.g., fish, via regression. Both approaches selected the XGBoost (extreme gradient boosting) ML algorithm for training the models. The validation results have demonstrated that while both methods have limitations (e.g., only features with MS/MS spectra can be considered) and are associated with uncertainty, they can effectively guide the NTS towards toxicologically relevant outcomes. Both tools enable a fully unsupervised and unbiased (by signal strength, the content of used databases, or what was identifiable) assessment of the whole detected space based on the toxicological relevance of the single features. The features predicted to be potentially toxic still have to be identified and verified, but as the number of potentially toxic compounds is 10–100 times smaller than the number of input signals, the analytical efforts can be minimized and concentrated on relevant toxicity drivers. While using the MS2Tox and MLinvitroTox outcomes for quantitative assessments is generally not recommended due to significant uncertainties from each modelling step, both can be applied for a stand-alone hazard-driven prioritization of unidentified compounds or in support of the traditional prioritization approaches based on signal intensity, frequency, or relevant trends in the data. These methods align well and could streamline HT-EDA studies focused on qualitatively identifying toxicity drivers.

In all of the above-mentioned approaches, feature priorization is performed post-acquisition using descriptors that refer to the presence of certain substructures or functional groups in the molecule. However, the structures most closely related to certain endpoints, many of which are well known and collected in databases such as ToxPrint [138], can be prioritized from data acquisition as well. An example of this on the fly structural alert search was carried out by Meekel et al. [139], to study their presence and prioritize potential toxicants from data acquisition. Integrating these toxicant prioritization strategies in both acquisition and NTS can facilitate the development of tailored endpoint-specific HT-EDA workflows that fulfil large-scale monitoring needs.

## Final recommendations and conclusions

The recent incorporation of effect-based methods into the regulatory framework of the WFD shows that the potential of bioanalytical tools for monitoring contaminants and assessing risks has been recognized, particularly in water bodies [140]. Currently, this recognition is limited to the use of specific bioassays to evaluate the presence of estrogenic hormones in water bodies, with the prospect of establishing effect-based trigger values in the future. If this implementation starts to be extended to a wider range of endpoints, it will lead us to question what are the factors responsible for exceeding such levels, so EDA can become a useful tool to complement these monitoring methods in regulations. However, a strategy as time- and work-consuming as conventional EDA is less suitable for aligning with regulatory demands. HT-EDA contributes towards eliminating this bottleneck while ensuring successful identification of the toxicity drivers, and thereby supporting the prioritization of chemicals for regulatory bodies.

Regarding bioassays, HT-EDA benefits greatly from the use of downscaled specific bioassays, that not only present very high sensitivity, but are also more compatible with toxicity prediction models to prioritize compounds in the NTS (so far mainly used for EDCs, but with ongoing progress for others such as mutagenicity or neurotoxicity). Similar to what has occurred with effect-based methods, if HT-EDA protocols for identifying toxicity drivers are eventually implemented in major regulations, it will likely begin with these endpoints. However, if HT-EDA aims to identify risk drivers in large-scale monitoring applications, focusing on just one endpoint might be wasteful. Therefore, two main short-term goals of HT-EDA should be to implement bioassay batteries to cover several specific endpoints, while bringing above-mentioned high-throughput in vivo bioassays into HT-EDA. The suggested endpoint coverage for effect-based methods, which depends on the sample type, is similarly applicable to HT-EDA. For instance, in wastewater monitoring, a recommended bioassay battery includes acute bacterial (Microtox), algal growth inhibition, estrogenic activity, glucocorticoid activity, xenobiotic metabolism (AhR and PXR), and genotoxicity assays [141, 142]. Studies by Zwart et al. and Houtman et al. [6, 17, 23], show the feasibility of using bioassay batteries in HT-EDA using those with the highest specificity among these recommendations. Yet, as emphasized in “Requirements, achievements, and challenges for bioanalytical tools in HT-EDA” section, toxicity assessment should not be limited to these and should be open to novel approaches.

**Fig. 4:**
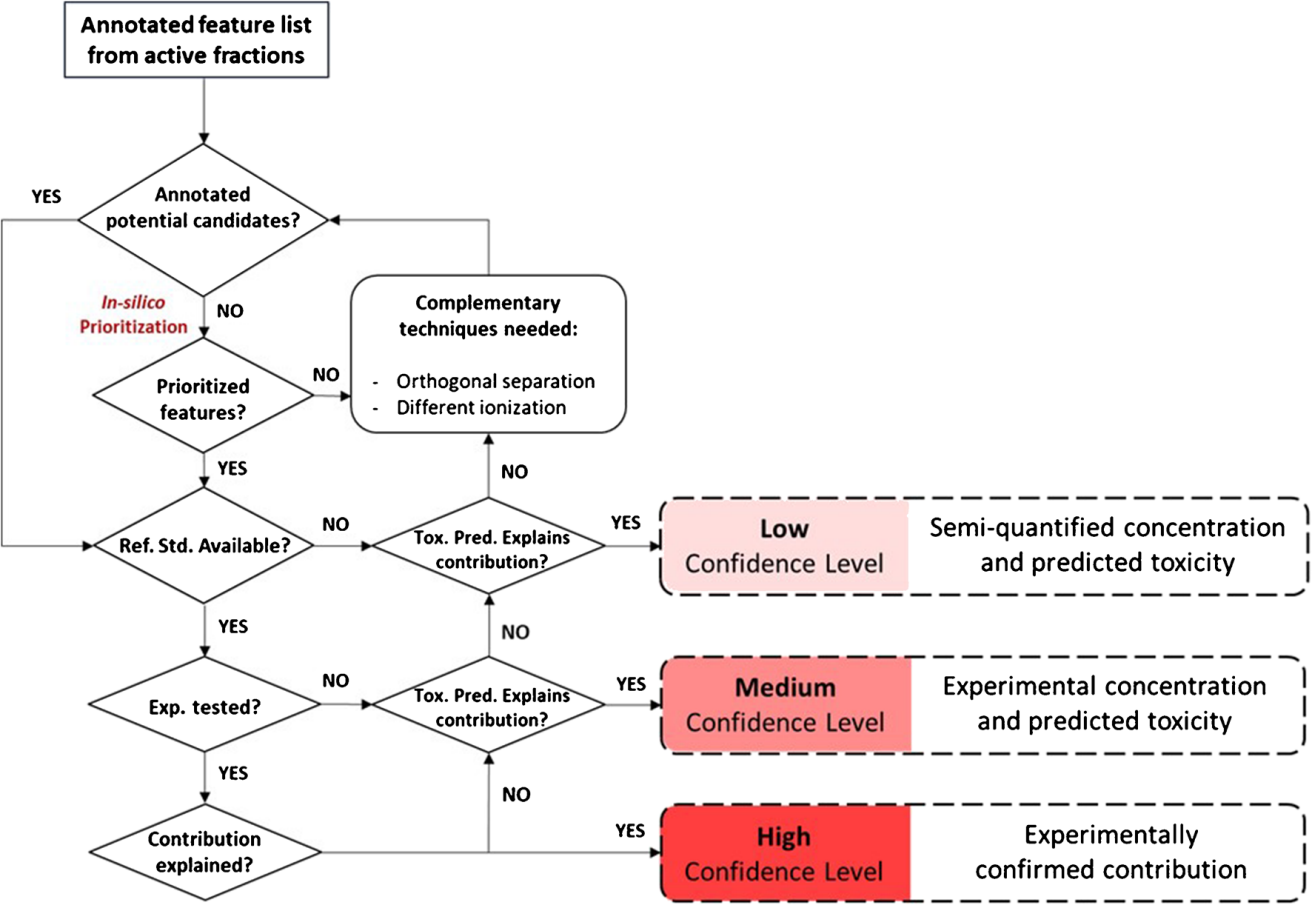
Recommended workflow scheme for toxicity driver identification in HT-EDA. The suggested confidence levels for toxicity contribution annotation are shown on the left

In each of the sections above, we have compiled and discussed novel tools that can aid in designing HT-EDA studies and ease the identification of toxicity drivers. However, applying them wisely is important to avoid unnecessary efforts. Thus, unless the sample type or the studied endpoint suggests otherwise, it is optimal to start with conditions covering the broadest range of compounds possible. For most cases, especially in aqueous samples, this involves using C18 RP columns for separation and ESI (+ and -) as the ionization technique. From this starting point, the first question to ask is whether, after analyzing toxic fractions, the feature list is sufficiently manageable to make the direct identification of toxicity drivers feasible without further prioritization (Fig. 4). Identifying some alleged toxicity drivers before prioritization might be possible for some specific endocrine disruption endpoints (e.g., finding testosterone, estrone, or other well-known androgenic or estrogenic compounds, respectively). However, this is uncommon for non-endocrine disruption endpoints, even if the separation resolution is high. If needed, in silico prioritization requires less effort (compared to applying orthogonal separation), making it advisable to consider it as the first option. Ideally, using an in vitro bioassay with a specific endpoint, toxicity-based prioritization would be the best choice, whether applied directly to features (the fastest option), to candidate structures, or both. Regardless of the prioritization method, a curated feature list will be obtained, with compounds identified at various confidence levels for which toxicity must be verified. If prioritization tools do not narrow down the list or the prioritized features do not explain the activity of the toxic fractions, two possible approaches can be explored: using orthogonal separation as an experimental prioritization technique or exploring complementary ionization techniques. Prioritization by experimental techniques such as orthogonal separation is less biased towards known-like structures, so may increase the chances of success if the risk drivers are novel toxicants. The second option would be preferable when we suspect potentially active compounds for the selected endpoint may have been overlooked under the previous conditions. When these tools cannot resolve sample toxicity, two options must be considered: either the compounds are not detectable in the method, or the mixture effects are responsible for toxicity rather than individual compounds. The latter is less likely with microfractionation, as narrower RT intervals result in having fewer features. Therefore, considering an alternative ionization source (such as APCI) could help expand the detectable chemical space and identify the responsible compounds. Still, no matter the chosen fractionation method, mixture effects should be checked by biotesting the mixture of identified compounds at the quantified concentrations if possible.

Although one of the aims of this review is to facilitate the implementation of HT-EDA in monitoring studies, it is important to be mindful of the associated costs and to use the approach smartly. Compared to conventional monitoring tools, whether chemical or toxicological, the use of HT-EDA involves more analytical or bioanalytical runs, greater material usage and more time due to demanding compound identification workflows. This directly results in higher costs, making it essential to apply HT-EDA only when truly necessary. Using HT-EDA on samples that show no activity in initial screenings, or whose activity can be explained by a few target compounds, is a misuse of resources. Therefore, it is advisable first to confirm that the activity of the sample is of concern (e.g., by comparing it with effect-based trigger values) and then check if the compounds found by the available targeted analysis can explain the biological activity of the sample.

Until now, the challenge in effect-based methods, specifically in (HT-)EDA, has centered around handling unknowns. The understanding of toxicity contribution has been limited to quantifiable and experimentally testable compounds, representing merely the tip of the iceberg. However, significant progress has been made, supported by the tools introduced in previous sections. Advancements in semi-quantification methods are progressing, and toxicity prediction tools are expanding to cover a growing number of endpoints and delivering predictions in the form of ECx or PNEC values. This implies that if our unknowns fall within the chemical space covered by the analytical technique and the application domain of the model, their contribution to total toxicity can be predicted. These predictions involve a higher degree of uncertainty, needing cautious use and, importantly, not replacing experimental assessment since bioanalytical tools are still the only way to realistically capture the toxicity of the whole iceberg. Whenever possible, uncertainty in the quantification and assessment of toxicity should be reported and extrapolated to the contribution. Besides broadening the scope of EDA by enabling semi-quantification of unknown contributions, these tools also provide additional evidence of candidate toxicity if HT-EDA is applied in an explorative manner.

Currently, all compounds contributing to toxicity move in a similar range of uncertainty in experimental measurements, but with the implementation of these new approaches, it may be necessary to distinguish the confidence with which the contribution is reported. To avoid misunderstandings and to seek a consensus that has worked well in other cases [143], we propose that future studies using these tools should classify contributing compounds into three levels (Fig. 4):

- High confidence level: Compounds that have been experimentally quantified and tested with reference standards.
- Moderate confidence level: Compounds that have been experimentally quantified with a reference standard but no experimental toxicity information is available, i.e., toxicity is predicted. The opposite case could also be considered at this level, but it is much less likely for practical reasons.
- Low confidence level: Compounds for which a semi-quantification without a reference standard is carried out and for which toxicity is predicted.

Certainly, there are numerous challenges to overcome before we can make HT-EDA a readily approachable and widespread tool. However, the innovations discussed throughout this review indicate that HT-EDA is on the right track to achieve this goal. In vitro bioassays with a specific endpoint are already well implemented in HT-EDA, performing robustly and seamlessly integrated with the new fractionation tools that reduce manual workload. In addition, the numerous computational tools that are emerging to facilitate the identification of toxicity drivers are creating new opportunities, but their value in HT-EDA applications remains to be proven. NTS is progressively gaining recognition in the analysis of environmental samples, and although there is still considerable ground to cover, the unknowns are becoming better known. With the support of the enhancements and implementations of computational tools, the toxicity of unknowns has started to unravel, marking also a significant turning point in (HT-)EDA. While establishing well-defined workflows for various environmentally relevant endpoints using receptor-based assays is a key focus of HT-EDA, it should not be limited to them. Other relevant endpoints should also be considered, for instance, by leveraging new approach methods (NAMs). The demonstrated potential of EDA to unravel toxicity drivers in complex mixtures is significant. However, the labor-intensive workload and uncertain success rates of traditional EDA have raised doubts about its scalability for large-scale applications. Recent advances and the range of tools in development are signalling a shift in this perception. No longer limited to isolated cases, HT-EDA must embrace these new computational and technological developments and get ready for monitoring applications.

## Supplementary Information

Below is the link to the electronic supplementary material.

Supplementary file1 (XLSX 82 KB)
